# Effect of regional versus general anesthesia on recurrence of non-muscle invasive bladder cancer: a systematic review and meta-analysis of eight retrospective cohort studies

**DOI:** 10.1186/s12871-023-02136-7

**Published:** 2023-06-13

**Authors:** Yulong Wang, Yuxuan Song, Caipeng Qin, Chunlong Zhang, Yiqing Du, Tao Xu

**Affiliations:** Department of Urology, Peking University People’s Hospital, Beijing, 100044 China

**Keywords:** General anesthesia, Regional anesthesia, Bladder cancer, Recurrence, Meta-analysis

## Abstract

**Background:**

Regional anesthesia appears to reduce cancer recurrence, but the optimal anesthesia modality for non-muscle invasive bladder cancer (NMIBC) were still under debate. Therefore, we sought to assess the effect of regional and GA only upon the recurrence and long-term prognosis of NMIBC through this meta-analysis.

**Methods:**

We performed an extensive literature search of PubMed, Embase, Web of Science, the Cochrane Library and China National Knowledge Infrastructure (up to October 30, 2022) to identify eligible articles on the possible impact of different anesthetic modalities for the recurrence rate of NMIBC.

**Results:**

Eight studies comprising 3764 participants, including 2117 subjects with RA and 1647 with GA, were finally enrolled. Cancer recurrence rate was significantly lower in subjects with RA than those with GA (RR 0.84, 95%CI 0.72–0.98, *P* = 0.03). We didn’t detect the differences between GA and RA in the time of recurrence (SMD 2.07, 95% CI -0.49–4.63, *P* = 0.11) and cancer progression (RR 1.14, 95%CI 0.71–1.84, *P* = 0.59). Results from subgroup analysis demonstrated that spinal anesthesia could significantly decrease the incidence of cancer recurrence in comparison with general anesthesia (RR 0.80, 95%CI 0.72–0.88, *P* < 0.001) and high-risk NMIBC patients who received RA tended to have less recurrence (HR 0.55, 95%CI 0.39–0.79, *P* = 0.001) than those receiving GA.

**Conclusions:**

RA, especially spinal anesthesia, may be effective in reducing the recurrence rate after transurethral resection of NMIBC. More prospective experimental and clinical studies are needed to validate our findings.

**Trial registration:**

INPLASY registration INPLASY2022110097.

**Supplementary Information:**

The online version contains supplementary material available at 10.1186/s12871-023-02136-7.

## Introduction

As the ninth most common cancer in the world [1], bladder cancer (BC) is the most common malignant tumor of the urinary system at present, with an estimated 550 000 new cases and 200 000 deaths worldwide in 2019 [2]. Non-muscle invasive bladder cancer (NMIBC) is the most common type of BC, accounting for about 75% of all cases in patients with BC [3]. For those with NMIBC, transurethral resection of bladder tumor (TURBT) is considered to be a prior treatment. Following TURBT surgery, the doctor will determine whether subsequent continuation of adjuvant treatment is necessary based on the histological origin of the BC and the TNM stage [4]. Recurrence and progression of NMIBC is common, with 50–70% of patients experiencing at least one recurrence within 5 years [3].

Perioperative factors, including surgical stress, blood transfusions, inhaled anesthetics, opioids, and hypothermia, all contribute to cancer recurrence through immunosuppression or cancer promotion [5–9]. The application of regional anesthesia (RA) has demonstrated the potential to suppress surgical stress as well as reduce opioid use, thereby possibly decreasing the occurrence of cancer recurrence [10, 11]. A retrospective study suggested for the first time that the use of paravertebral anesthesia may improve recurrence-free survival for patients with breast cancer [12]. Unlike other malignancies, types of anesthesia adopted during TURBT seemed to not influence the recurrence rate of BC [13], and some recent studies supported this conclusion [14, 15]. However, some researchers have linked the use of spinal anesthesia to a lower rate of recurrence of NMIBC [16–18].

Recently, there have been many studies [14–21] exploring the association between anesthesia methods and recurrence of the NMIBC after TURBT. However, the inconsistent conclusions and limited subjects of these articles reduce their credibility of the evidence. A previous study comparing the impact of RA only with general anesthesia (GA) upon cancer recurrence included only three researches of BC and significant heterogeneity was detected among the selected articles in assessing the final results [22]. The optimal modality of anesthesia for NMIBC remains controversial. Therefore, we sought to compare and assess the effect of regional and GA only upon the recurrence and long-term prognosis of NMIBC through this more precise meta-analysis.

## Methods

This systematic review and meta-analysis was reported in accordance with the Cochrane Handbook for Systematic Reviews and Interventions and the Preferred Reporting Items for Systematic Reviews and Meta-analyses (PRISMA) [23]. We registered our systematic review protocol in the INPLASY (INPLASY2022110097) on November 20,2022.

### Literature search strategy

We performed an extensive literature search on the PubMed, Embase, Web of Science, the Cochrane Library and China National Knowledge Infrastructure (up to October 30, 2022) to identify eligible articles on the possible impact of different anesthetic modalities for the recurrence rate of NMIBC. We adopted a search pattern of subject terms combined with their respective free terms. Among the subject terms were “urinary bladder neoplasms”, “regional anesthesia” and “general anesthesia”. The languages of the included studies were English and Chinese. Other possible studies were collected through searching the references of the enrolled literature.

### Eligibility and exclusion criteria

Enrolled studies met these criteria: (1) The study subjects were diagnosed with NMIBC and received TURBT with RA or GA; (2) Subjects in the intervention group underwent RA only during surgery; (3) Subjects in the control group underwent GA during surgery; (4) Studies compared cancer recurrence, time to recurrence, progression as well as survival rates between the two groups. (5) The type of literature was a cohort study or a randomized clinical trial. The main exclusion criteria were: (1) other types of cancers; (2) patients with combined multiple anesthetic modalities; (3) no comparison of cancer recurrence rates between the control and intervention groups after surgery; (4) Letters, reviews, comments, author responses and case report studies.

### Data collection

The results of the database searches were conducted by two researchers independently and data was collected from every of the eligible articles. When disagreements arose, a third reviewer would join the discussion and reach agreement. Features of the literatures were collected (including first author’s name, publication year, country, ethnicity, study types and number of subjects), basic patient information (including clinicopathological stage, ASA score, mode of anesthesia).

### Bias and methodological quality assessment

We assessed eligible cohort studies for risk of bias and quality assessment utilizing the Newcastle–Ottawa Scale (NOS) [24]. The scale evaluates the literature in terms of the appropriateness of study population selection, comparability between groups, and clarity and adequacy of exposures and outcomes. High quality studies generally require a score of more than 7 on the scale.

### Statistical analysis

We applied relative risks (RR) and hazard ratios (HR) to compare the different anesthetic modalities in relation to the recurrence rate, progression and overall survival of NMIBC. Standardized mean differences (SMD) were adopted to compare the differences in time to NMIBC recurrence between the two groups. We applied their 95% confidence intervals (95%CI) for final comparison of the pooled results. We also adopted random or fixed effects models to calculate RR, HR, and SMD. Heterogeneity among different studies was detected through the I^2^ as well as Chi-squared test results. Fixed effect model (FEM) was used when *P* > 0.05 for Chi-squared test or I^2^ < 50% [25]; Alternatively, random effect model (REM) [26] was applied. Besides, different types of RA and the risk of BC were adopted for subgroup analyses to explore their possible influence on the pooled outcomes. We also used sensitivity analyses to evaluate the robustness and reliability of the outcomes by removing every enrolled literature in turn. Begg's test as well as Egger's test was adopted to measure publication bias. We conducted our meta-analysis with Stata software (version 12.0) and Review Manager (version 5.3, Copenhagen: Nordic Cochrane Centre, The Cochrane Collaboration, 2014).

## Results

### Literature selection and characteristics of the enrolled articles

**Fig. 1:**
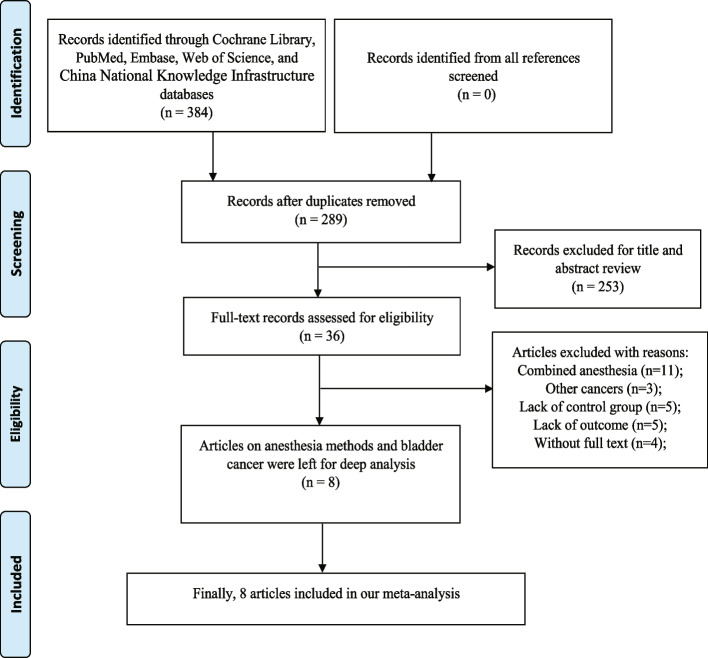
Flow chart of the process of literature search and study enrollment

We collected 384 records based on the initial search strategy. After removing duplicate articles, 289 documents were retained. By scanning the title or abstract of potential articles, 253 studies were excluded and 36 were eventually included for reading in full. According to our meta-analysis inclusion and exclusion criteria, eight studies comprising 3764 participants, including 2117 patients with RA and 1647 patients with GA, were finally enrolled. The process of literature search and study enrollment was displayed in Fig. 1. All the literatures were retrospective cohort studies. Seven articles were single-center, while one [18] was multicenter. The specific methods of RA included spinal anesthesia and epidural anesthesia, and the anesthetics used included lidocaine, ropivacaine, bupivacaine and midazolam. Total intravenous anesthesia and inhalational anesthesia were regarded as GA. Anesthetics used included propofol remifentanil, fentanyl, sevoflurane, nitrous oxide, etomidate, vecuronium, rocuronium, cis-atracurium, thiopental and atracurium. The features of the enrolled studies are present in Table 1.

**Table 1:** Main clinical characteristics of the included studies

First Author	Year	Country	Stage	Study design	Total	RA				GA				Follow-up
						Agents	Number	Age	Gender(M/F)	Agents	Number	Age	Gender(M/F)	(months)
Cheng Luo	2020	China	NMIBC	Retrospective single center	302	Marcaine	182	60^a^	141/41	Propofol, Remifentanil Sevoflurane	120	60^a^	98/22	33.5
Dale Jang	2016	Korea	T1-T2	Retrospective single center	161	Bupivacaine, Lidocaine	137	62.4 ± 10.8	107/30	Propofol, Etomidate Vecuronium, Rocuronium	24	67.5 ± 9.0	17/7	60
Ruifeng Xue	2022	China	NMIBC	Retrospective single center	528	Bupivacaine, Lidocaine, Ropivacaine	264	56.62 ± 13.809	239/25	Cis-atracurium, Fentanyl, Propofol, Remifentanil, Sevoflurane	264	58.54 ± 11.968	229/35	36
Sang Won Lee	2022	Korea	NMIBC	Retrospective single center	1164	NA	582	66.75 ± 6.09	460/122	NA	582	66.50 ± 6.06	460/122	52.6
Tingting Wang	2019	China	NMIBC	Retrospective single center	388	Bupivacaine Midazolam	136	69.27 ± 13.00	207/45	Propofol, Remifentanil, Rocuronium	252	62.09 ± 9.00	100/36	35.6
Woo-Jong Choi	2017	Korea	NMIBC	Retrospective single center	690	Bupivacaine, Midazolam	534	62 ± 12	436/98	Sevoflurane, Thiopental, Rocuronium, Atracurium, Nitrous oxide	156	61 ± 13	124/32	35
Yuri Koumpan	2018	Canada	NMIBC	Retrospective single center	231	NA	135	71.72 ± 10.57	109/26	NA	96	65.45 ± 10.86	77/19	65
Yuto Baba	2021	Japan	NMIBC	Retrospective multicenter	300	Bupivacaine	147	75^b^	115/32	Fentanyl, Propofol, Sevoflurane	153	75^b^	129/24	46.5

*NMIBC* non-muscle invasive bladder cancer, *RA* regional anesthesia, *GA* general anesthesia, *M/F* male/female, *NA* not available

^a^RA: ≥ 60:114, < 60:68; GA: ≥ 60:80, < 60:40; ^b^RA: ≥ 75:37, < 75:110;GA: ≥ 75:64, < 75: 89

### Bias and quality assessment

The bias and quality assessment of the eight enrolled articles is demonstrated in Supplemental Table 1. All the eligible studies in our meta-analysis were well-represented and exposed. Three studies [14, 15, 17] were propensity matched, and the comparability was good. However, four studies [16, 18–20] have significant differences in the age of patients receiving RA and GA, which may induce confounding bias and thus score poorly on comparability. The mean follow-up time was around three years in all included studies. Overall, all eight included studies were of high quality.

### Meta-analysis results

#### Cancer recurrence rate

**Fig. 2:**
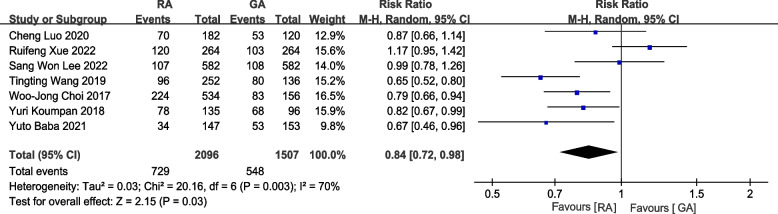
Forest plot of cancer recurrence rate (RA vs GA)

Seven studies with 3603 patients reported postoperative cancer recurrence rates [14–18, 20, 21]. The pooled results indicated that the postoperative cancer recurrence rate was significantly lower among the subjects with RA than those who underwent GA. (RR = 0.84,95%CI = 0.72–0.98, *P* = 0.03, Fig. 2).

### Time to recurrence

**Fig. 3:**
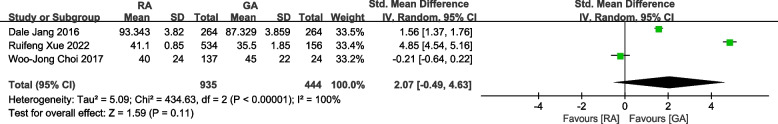
Forest plot of time to recurrence (RA vs GA)

Three studies, including 1379 patients, reported time to recurrence [14, 17, 19]. The duration of cancer recurrence after transurethral resection of BC did not differ significantly among subjects with RA or GA (SMD = 2.07, 95% CI = -0.49–4.63, *P* = 0.11, Fig. 3).

### Progression

**Fig. 4:**
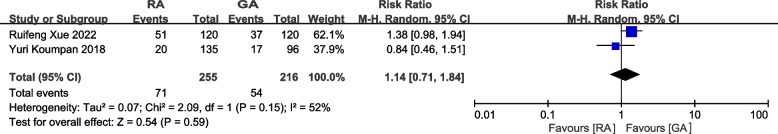
Forest plot of progression (RA vs GA)

Two studies, comprising 300 subjects, compared cancer progression between two groups [14, 16]. No significant association was found between RA or GA and the progression of NMIBC (RR = 1.14, 95%CI = 0.71–1.84, *P* = 0.59, Fig. 4).

### Subgroup analysis of type of RA, GA and risk of NMIBC

When subgroup analyses [14–18, 20] were performed based on the specific types of RA [14–18, 20], we found that spinal anesthesia significantly reduced the recurrence of NMIBC (RR = 0.80, 95%CI = 0.72–0.88, *P* < 0.001, Supplemental Fig. 1). In contrast, there was no significant advantage in cancer recurrence for patients with epidural anesthesia in comparison with those who received GA (RR = 1.17, 95%CI = 0.95–1.42, *P* = 0.14, Supplemental Fig. 1). When the different types of general anesthesia are further considered, RA has obvious advantages in reducing the recurrence rate of NMIBC, compared with both inhalational anesthesia (RR = 0.78, 95%CI = 0.68–0.90, P = 0.0007, Supplemental Fig. 2) and total intravenous anesthesia (RR = 0.65, 95%CI = 0.52–0.80, *P* < 0.0001, Supplemental Fig. 2). We also conducted the subgroup analysis based on risk stratification of NMIBC [15, 16, 18], Similarly, the adoption of RA may lead to less recurrence in high-risk NMIBC (HR = 0.55, 95%CI = 0.39–0.79, *P* = 0.001, Supplemental Fig. 3). However, different anesthetic modalities had no obvious impact on the recurrence of low-risk NMIBC (HR = 0.95, 95%CI = 0.58–1.53, *P* = 0.82, Supplemental Fig. 3).

### Sensitivity analyses and publication bias detection

We applied the sensitivity analysis to evaluate the stability of all aggregated outcomes by sequentially excluding every single article. As it could conclude from Supplemental Fig. 4, the overall results of our literature were stable, with no individual study obviously altering the final results. Egger's test and Begg's test were applied to monitor for possible publication bias among the enrolled studies. The results detected no publication bias, which further supported the credibility of the meta-analysis. (*P* > 0.05, Supplemental Fig. 5).

## Discussion

Patients with NMIBC who received RA tended to be less likely to present with recurrence than those with GA. Notably, there was no obvious effect of the two anesthesia methods on the time to recurrence, survival and progression rates of subjects with NMIBC. A further subgroup analysis found that spinal anesthesia was more effective than epidural anesthesia in reducing cancer recurrence rate in comparison with GA. Interestingly, we also discovered that RA significantly decreased the recurrence of NMIBC, whether compared with total intravenous anesthesia or inhalational anesthesia. Similarly, cancer recurrence in high-risk NMIBC was significantly reduced with RA. In contrast, recurrence in low-risk patients was not found to be related to the types of anesthesia.

GA is performed with intravenous anesthetics, inhaled anesthetics, or a combination of the two. Volatile anesthetics have been proven to suppress the immune system and promote inflammatory responses, and therefore affects the survival of tumor cells, including by regulating gene expression in immune cells [27, 28]. For example, sevoflurane inhibits the function of T lymphocytes as well as increases the production of hypoxia-inducible factor (HIF) and insulin-like growth factor, thereby promoting cancer growth [29–31]. On the contrary, natural killer cells are activated and cancer growth is reduced owing to the regional anesthetics, such as lidocaine [29]. Surgical stress also inhibited NK cell activity [32] and increases the production of some of the ILs associated with cancer progression, among which the elevated level of IL-6 promotes the angiogenesis and progression of BC [33] and IL-8 is associated with tumor recurrence [34]. Regional anesthetics have been shown to reduce surgical pressure, and a diminished stress response could also decrease the occurrence of immunosuppression [10, 35]. Moreover, high-risk NMIBC has a longer operation time and tremendous surgical pressure. Thus, this may explain the advantage of RA in decreasing the recurrence of high-risk NMIBC. RA also reduces the dose of opioids used. Opioids can prevent natural killer cells from functioning properly and can also inhibit immune pathways of immunoglobulin secretion and cytokine release, therefore promoting the recurrence of tumors [36].

Among patients with muscle-invasive bladder cancer, spinal anesthesia combined with GA achieved opioid-sparing results without significant improvement in final oncological outcomes [37]. Some population-based analyses also found that using epidural anesthesia for radical cystectomy did not improve cancer-specific or overall survival [38, 39]. Interestingly, when sufentanil was adopted in epidural anesthesia, a higher recurrence rate and shorter disease-free survival was found among BC patients undergoing radical surgery. This may be the result of the sufentanil itself or the adoption of this drug which in turn increases the total amount of morphine equivalents absorbed by the patients, given the immunosuppressive effects present with opioids [40]. However, these studies all used a combination of RA and GA. This may be why RA does not seem to improve outcomes in cancer patients: RA applied in addition to GA is insufficient to counteract the adverse effects of GA. In addition, it should also be considered that the malignant degree of MIBC is significantly higher. Compared with pathological stage, lymph node invasion and neoadjuvant chemotherapy, the influence of an anesthesia regimen on patients with MIBC may be insignificant.

When the types of regional anesthesia used was further considered, spinal anesthesia during surgery in patients with NMIBC is effective in reducing the recurrence rate. Spinal anesthesia suppresses cancer growth by inhibiting sodium channels, which forces cancer cells to re-express voltage-gated sodium channels and reduces metastatic cells activity [41]. In contrast, one cohort study [14] showed that the adoption of epidural anesthesia failed to obviously reduce cancer recurrence. The studies [38, 39] mentioned above focusing on MIBC also used epidural anesthesia and did not significantly improve the prognosis of patients. It has even been claimed that the combination of epidural anesthesia reduces the disease-free recurrence rate and cancer-specific mortality of patients. In addition, different types of GA also seem to influence postoperative patient outcomes. Compared with intravenous anesthesia, inhalational anesthesia for radical resection promotes the potential of distant recurrence as well as favors a higher tumor pathological stage [42]. Similarly, some studies also confirmed longer disease-free survival in subjects that received propofol than those with sevoflurane and opioids [43, 44]. This could be explained by the reason that propofol does not inhibit natural killer cell function but inhibits cancer cells from spreading, invading, or surviving [44, 45].

According to a prospective clinical study, epidural anesthesia during surgery does not improve their disease-free survival [46]. Similarly, a meta-analysis that included six prospective clinical trials came to the conclusion that the combination of RA and GA did not decrease cancer recurrence after tumor resection [47]. The anesthesia approach for malignant tumor surgery is still controversial, and more randomized clinical trials should be conducted to find and explore the best anesthetic methods for different types of cancers. Unlike the results of the previous meta-analysis [48, 49], this article failed to detect a relationship between RA and cancer progression rates. This could be due to the low progression rate of early BC [4], and the influence of anesthesia methods on the prognosis of BC is more reflected in the recurrence rate of cancer.

Our study is the first meta-analysis of anesthesia methods for NMIBC. The included studies used only RA or GA, excluding the impact of combined RA and GA application on outcomes. However, although we conducted as thorough a literature search as possible, all the enrolled researches were retrospective studies, which reduced the credibility of the conclusions to some extent. In addition, some studies were not propensity-matched, which may also cause confounding bias. Although significant heterogeneity was detected in the included studies, heterogeneity was reduced after subgroup analysis. However, reduced heterogeneity of high-risk patients did not seem to exclude the effect of chance due to the limited inclusion of literature. Therefore, large prospective clinical studies and meta-analyses are required to further explore these questions.

## Conclusion

RA, especially spinal anesthesia, may be effective in reducing the recurrence rate after transurethral resection of NMIBC. More prospective experimental and clinical studies are needed to validate our findings.

## Supplementary Information

**Additional file 1:** **Supplemental Table 1.** The risk assessmentof the included studies.

**Additional file 2:** **Supplemental Fig 1.** Subgroup analysis based on types of RA (epiduralanesthesia/spinal anesthesia : GA).

**Additional file 3:** **Supplemental Fig 2.** Subgroup analysis based on types of GA (RA :inhalational anesthesia/total intravenous anesthesia).

**Additional file 4:** **Supplemental Fig 3.** Subgroupanalysis based on the risk of NMIBC.

**Additional file 5:** **Supplemental Fig 4.** Sensitivity analysis of anesthesia type.

**Additional file 6:** **Supplemental Fig 5. **Begg’sfunnel plot for publication bias test under cancer recurrence rate (RA vs. GA).

## Data Availability

The datasets generated and analyzed during the current study are available from the corresponding author upon reasonable request.

## Abbreviations

BC: Bladder cancer
CI: Confidence interval
GA: General anesthesia
HR: Hazard ratio
NMIBC: Non-muscle invasive bladder cancer
TURBT: Transurethral resection of bladder tumor
RA: Regional anesthesia
RR: Relative risk
SMD: Standardized mean difference
