# Supplementary material for: Effect of regional versus general anesthesia on recurrence of non-muscle invasive bladder cancer: a systematic review and meta-analysis of eight retrospective cohort studies
Source: BMC Anesthesiol. 2023 Jun 13;23:201. doi: 10.1186/s12871-023-02136-7 (PMC10262544; doi:10.1186/s12871-023-02136-7)
Supplement: Supplementary file 1 — Additional file 1: Supplemental Table 1. The risk assessmentof the included studies. [file 12871_2023_2136_MOESM1_ESM.docx]

| **First Author** | **Year** | **Selection** | | | | **Comparability** | **Outcome** | | | **Total Scores** |
| --- | --- | --- | --- | --- | --- | --- | --- | --- | --- | --- |
|  |  | Representativeness of the exposed cohort | Selection of the non-exposed cohort | Ascertainment of exposure | Outcome of interest was not present at start of study | Comparability of cohorts on the basis of the design or analysis | Assessment of outcome | Follow-up long enough for outcomes to occur | Adequacy of follow up of cohorts |  |
| **Cheng Luo** | **2020** | * | * | * | * | * | * | * | * | 8 |
| **Dale Jang** | **2016** | * | * | * | * |  | * | * | * | 7 |
| **Ruifeng Xue** | **2022** | * | * | * | * | ** | * | * | * | 9 |
| **Sang Won Lee** | **2022** | * | * | * | * | ** | * | * | * | 9 |
| **Tingting Wang**  **Woo-Jong Choi**  **Yuri Koumpan**  **Yuto Baba** | **2019**  **2017**  **2018**  **2021** | *  *  *  * | *  *  *  * | *  *  *  * | *  *  *  * | ** | *  *  *  * | *  *  *  * | *  *  *  * | 7  9  7  7 |

**Supplemental Table 1**: The risk assessment of the included studies
