# Supplementary figures and images for: Effect of regional versus general anesthesia on recurrence of non-muscle invasive bladder cancer: a systematic review and meta-analysis of eight retrospective cohort studies
Source: BMC Anesthesiol. 2023 Jun 13;23:201. doi: 10.1186/s12871-023-02136-7 (PMC10262544; doi:10.1186/s12871-023-02136-7)

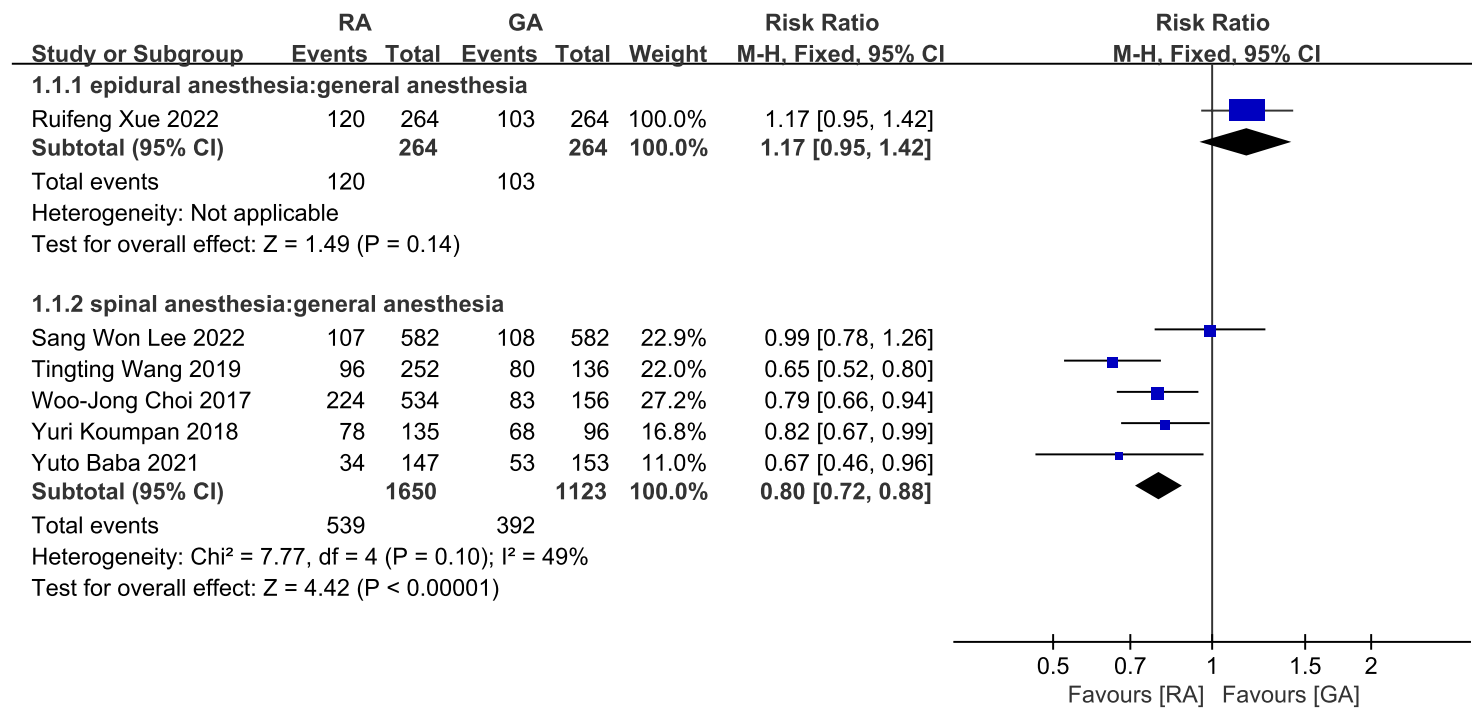

Supplement: Supplementary file 2 — Additional file 2: Supplemental Fig 1. Subgroup analysis based on types of RA (epiduralanesthesia/spinal anesthesia : GA). [file 12871_2023_2136_MOESM2_ESM.pdf]

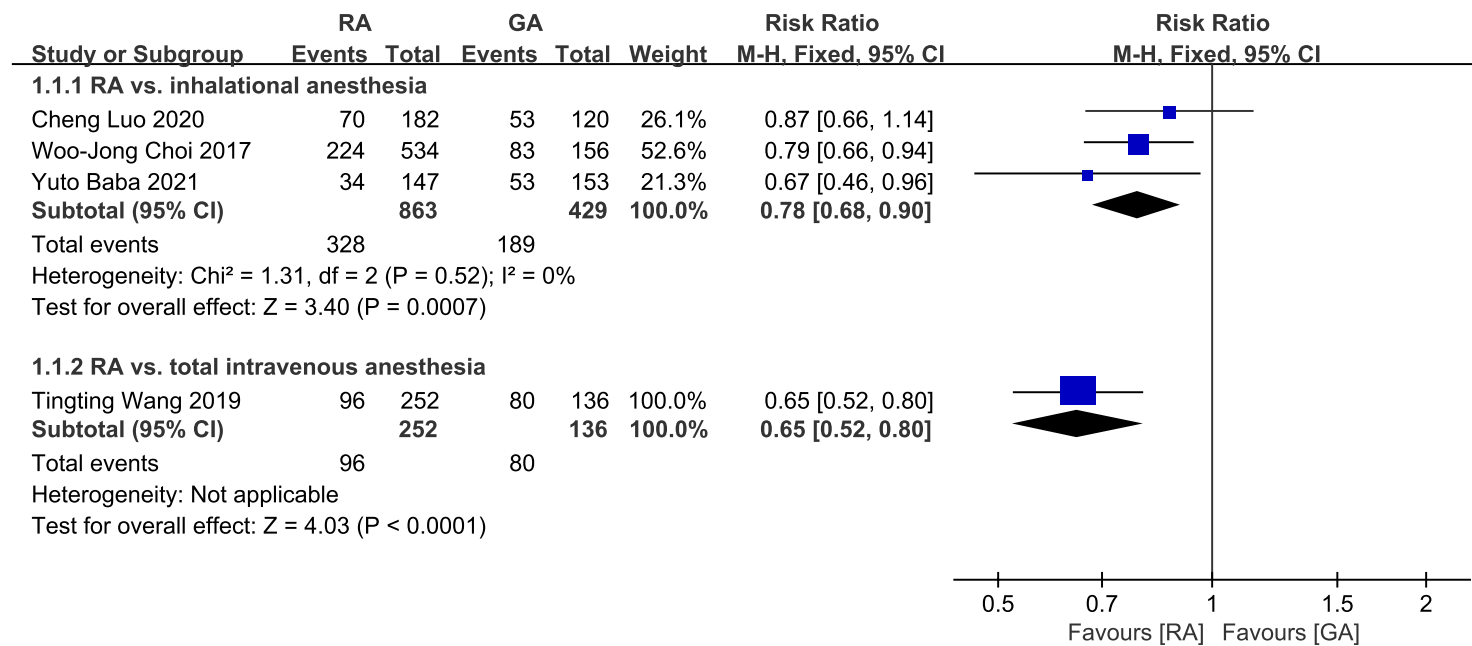

Supplement: Supplementary file 3 — Additional file 3: Supplemental Fig 2. Subgroup analysis based on types of GA (RA :inhalational anesthesia/total intravenous anesthesia). [file 12871_2023_2136_MOESM3_ESM.pdf]

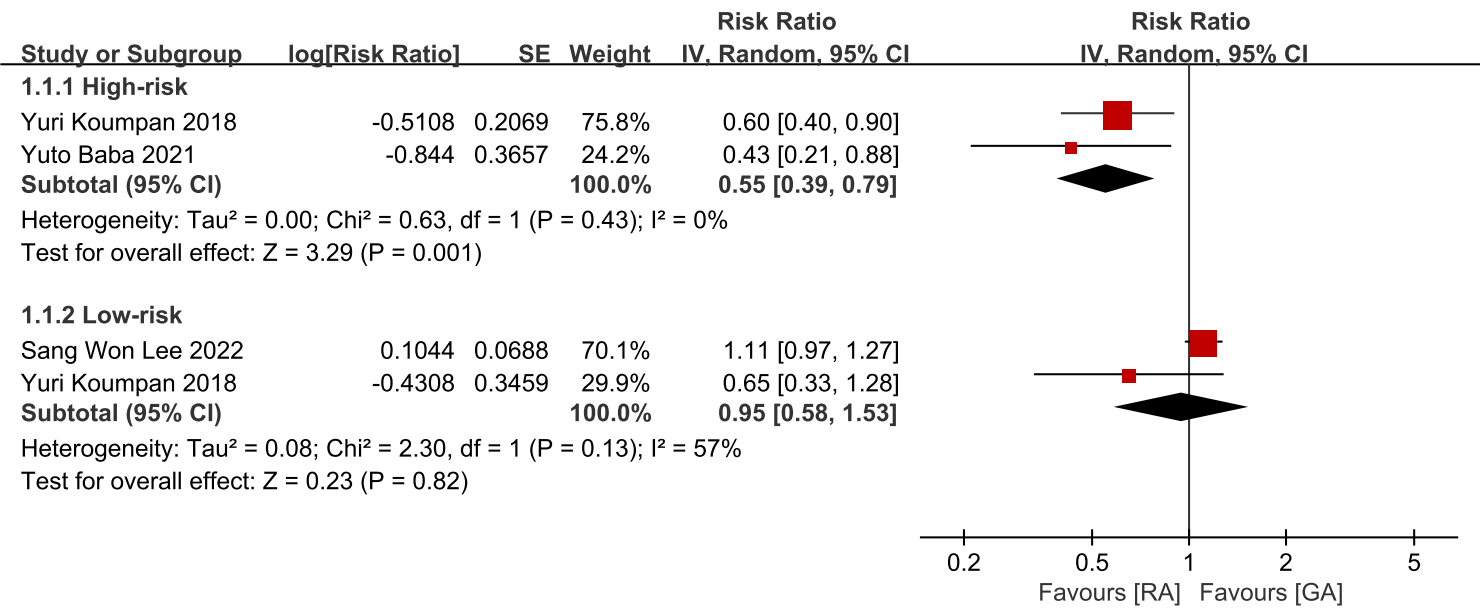

Supplement: Supplementary file 4 — Additional file 4: Supplemental Fig 3. Subgroupanalysis based on the risk of NMIBC. [file 12871_2023_2136_MOESM4_ESM.pdf]

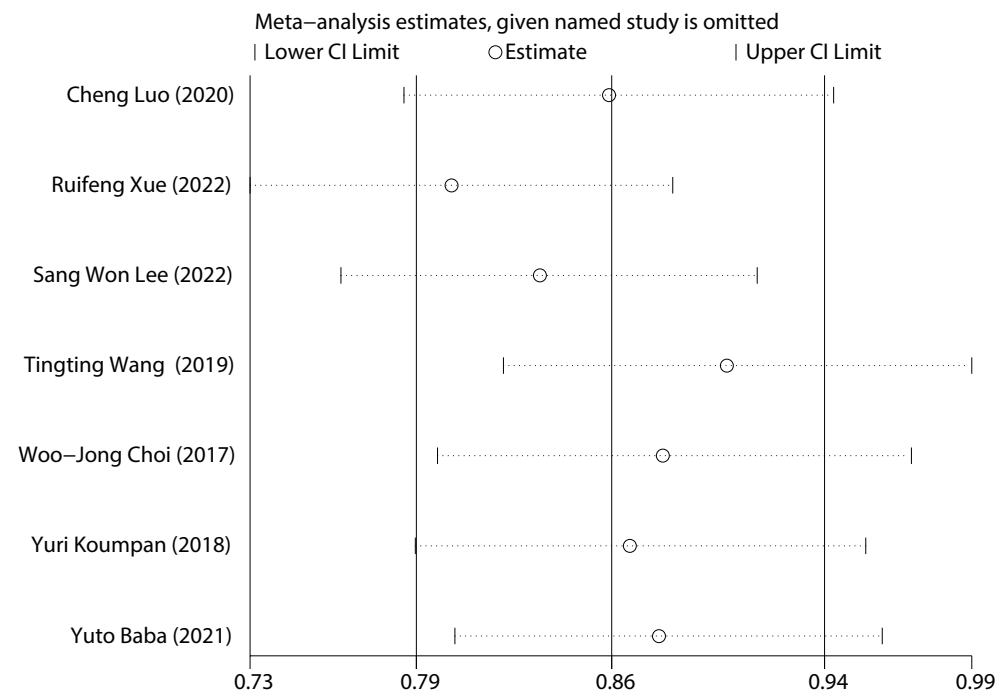

Supplement: Supplementary file 5 — Additional file 5: Supplemental Fig 4. Sensitivity analysis of anesthesia type. [file 12871_2023_2136_MOESM5_ESM.pdf]

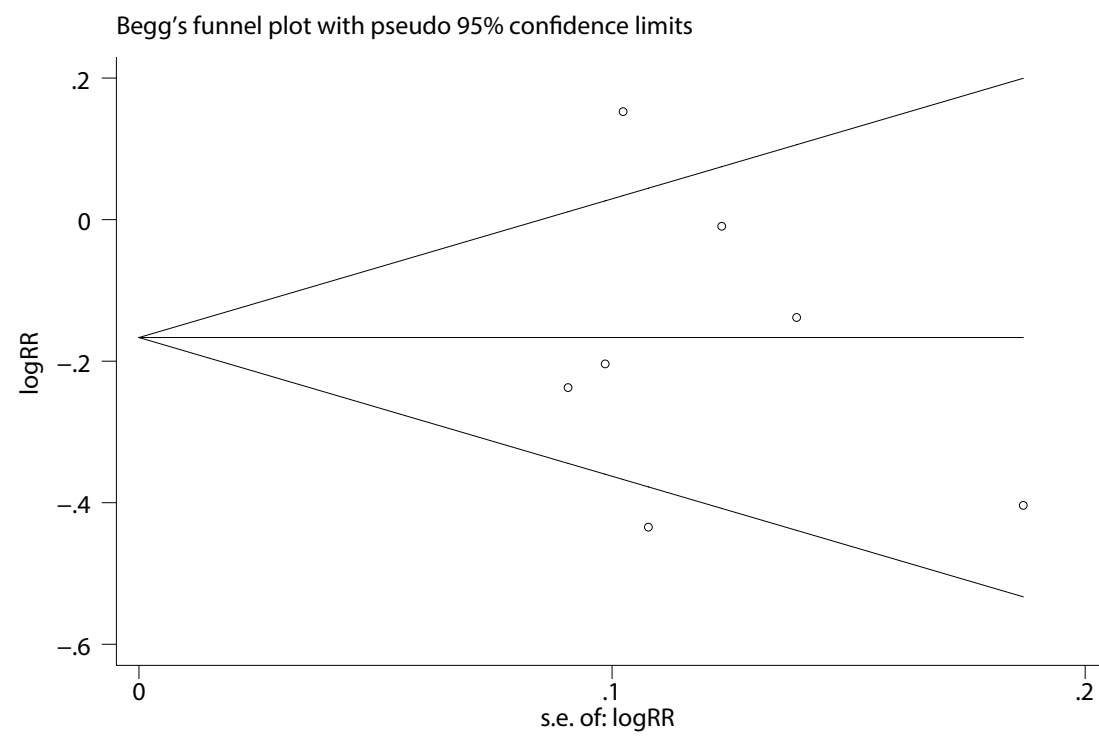

Supplement: Supplementary file 6 — Additional file 6: Supplemental Fig 5. Begg’sfunnel plot for publication bias test under cancer recurrence rate (RA vs. GA). [file 12871_2023_2136_MOESM6_ESM.pdf]
